# Conceptualizing Physicians’ Roles in Addressing Intimate Partner Violence: A Critical Discourse Analysis of Resources for Canadian Physicians

**DOI:** 10.1177/10778012221114922

**Published:** 2022-08-22

**Authors:** Alice Cavanagh, Melissa Kimber, Harriet L. MacMillan, Stacey A. Ritz, Meredith Vanstone

**Affiliations:** 1 62703 McMaster University, Hamilton, Canada

**Keywords:** medical education, intimate partner violence, medicalization

## Abstract

Resources addressing intimate partner violence (IPV) play a role in shaping how physicians conceptualize and perform their roles in caring for affected patients. This study combines environmental scanning with critical discourse analysis (CDA) to parse how roles of physicians were represented in 28 education materials and policy documents about IPV, taking the Canadian training milieu as an example. We developed a cyclical model of three core physician roles in addressing IPV—learning about IPV, identifying patients experiencing IPV, and responding to patients’ disclosures of IPV. The construction of these physician roles is suggestive of an ongoing process of medicalization of IPV.

## Introduction

Intimate partner violence (IPV) is defined by the World Health Organization (WHO) as “behaviour within an intimate relationship that causes physical, psychological or sexual harm to those in the relationship” (World Health Organization [WHO], 2012). Around the world, 30% of ever-partnered women report having experienced IPV at least once in the course of their lives (WHO, 2021). The health impacts associated with IPV are profound, wide-ranging, and economically significant. IPV is associated with acute injuries stemming from physical trauma and chronic stress-related mental and physical health conditions that endure over the life course, leading to increased health care utilization and spending (Bidarra et al., 2016; Bonomi et al., 2006; Campbell, 2002; Henry et al., 2018; Jordan et al., 2010; McTavish et al., 2016; Peterson et al., 2018; Stewart & Vigod, 2017. Fortunately, research evidence suggests that timely, informed support from a health care provider can play an important role in mitigating both immediate and longer-term sequelae of IPV and gaining access to other supports (WHO, 2013). In view of this, the WHO recommends that education about IPV be incorporated across all stages of medical training (WHO, 2013) but the state of progress toward realizing this goal remains unclear. Indeed, research demonstrates that physicians’ lack of knowledge about IPV can discourage patients from seeking help in health care settings (Caralis & Musialowski, 1997; McCauley et al., 1998; Rodriguez et al., 1996) and inhibit practitioners from raising concerns with patients (Saletti-Cuesta et al., 2018).

Research that addresses the scope of training and education that physicians receive related to IPV is largely descriptive, quantifying or qualifying coverage in undergraduate or postgraduate medical education (UGME or PGME) (Baker, 1995; Davidson et al., 2001; Frank et al., 2006; Sammut et al., 2019; Short et al., 1998; Wathen et al., 2009; WHO, 2013) or evaluating outcomes stemming from specific education programs for practicing physicians (Baker, 1995; Committee on Accreditation of Canadian Medical Schools, 2021; Davidson et al., 2001; Frank et al., 2006; Harris et al., 2009; Sammut et al., 2019; Short et al., 1998; Short et al., 2006; WHO, 2013). Free educational materials related to IPV are both available and accessible online, and previous research has demonstrated the growing utilization of similar resources among physicians (MacWalter et al., 2016; van der Keylen et al., 2020). As of yet, however, no study has sought to analyze the landscape of free training materials related to IPV that are available to physicians online, despite evidence that the use of online resources can impact physicians’ perceptions of their knowledge and readiness to respond to patients affected by IPV (Kalra et al., 2021). To address this gap in the literature, we conducted a critical discourse analysis (CDA) of contemporary IPV resources for practicing physicians. Our objective was to examine how physicians’ roles in addressing IPV were represented across these education materials, taking the Canadian training milieu as an example.

## Methods

### Study Design and Methodology

This research was carried out as part of the first phase of a larger sequential mixed method research project examining physicians’ and social workers’ preferences regarding education about IPV and other forms of family violence (Kimber et al., submitted). The current project addresses one component of this project, focused only on physicians. We conducted an environmental scan to ensure the IPV resources we examined in our CDA represented the full breadth of those available to physicians. Environmental scanning is a flexible, yet rigorous, approach to “seeking, gathering, interpreting and using information” (Charlton et al., 2019) that is well-suited to contexts where data span a wide range of nontraditional sources. For this study, because training and education materials are not typically published in peer-reviewed journals, a search strategy using academic databases was not viable. Instead, we used environmental scanning methodology to devise a systematic search strategy targeting organizations involved in resource development.

### Search Procedure and Inclusion Criteria

We began our search by compiling a list of 150 Canadian organizations with potential involvement in developing resources related to family violence for physicians (Table 1). This list was reviewed and revised by a consulting group of Canadian practitioner-scientists with expertise in family violence to ensure comprehensiveness before searching began. These individuals were engaged as part of the national advisory board for the larger project and represent a variety of health professions and academic disciplines (Kimber et al., submitted). We used the site search function on Google.com to search each organization’s website, combining clusters of search terms related to IPV or child maltreatment, with another cluster relating to various resource formats (Table 2). The progress of these searches, as well as any identified resources, were tracked by team members using shared online spreadsheets (Appendix 1). Prospective resources were reviewed by two team members to verify their eligibility and their areas of focus and intended audience. The final list of identified resources was recirculated to the expert consulting group to ensure its comprehensiveness. Searching began on April 7, 2020 and finished 5 months later on July 2, 2020. The present study is a secondary analysis of the subset of resources identified in the scan that addressed IPV for an audience of physicians (Table 3).

**Table 1.** List of Organizations Initially Identified for Website Search.

Physician colleges	Physician associations	Federal government	Provincial/territorial governments	Specialty-specific organizations	Research-oriented organizations	Service-oriented organizations	Policy-oriented organizations
Federal or provincial/ territorial regulatory bodies that govern medical practice.	Federal or provincial/ territorial medical advocacy organizations that represent physician interests.	Departments or agencies of the federal government of Canada.	Departments or agencies of any of the provincial or territorial governments.	Advocacy organizations that represent physicians in psychiatry, pediatrics, emergency medicine, or family medicine.	Organizations with a primary focus on producing research, at or apart from, postsecondary institutions.	Organizations with a focus on service provision or supporting service provision.	Organizations with a primary focus on policy research or advocacy.
- The Royal College of Physicians and Surgeons of Canada - College of Family Physicians of Canada *Alberta* - College of Physicians & Surgeons - College of Family Physicians *British Columbia* - College of Physicians & Surgeons - College of Family Physicians *Manitoba* - College of Physicians & Surgeons - College of Family Physicians *New Brunswick* - College of Physicians & Surgeons - College of Family Physicians *Newfoundland & Labrador:* - College of Physicians & Surgeons - College of Family Physicians *Nova Scotia* - College of Physicians & Surgeons - College of Family Physicians *Prince Edward Island* - College of Physicians & Surgeons - College of Family Physicians *Ontario* - College of Physicians & Surgeons - College of Family Physicians *Québec* - Collège des médecins - Collège québécois des médecins de famille *Saskatchewan* - College of Physicians & Surgeons - College of Family Physicians - Yukon Medical Council	- Canadian Medical Association - Doctors of BC - Alberta Medical Association - Saskatchewan Medical Association - Doctors Manitoba - Ontario Medical Association - New Brunswick Medical Society - Doctors Nova Scotia - Medical Society of Prince Edward Island - Newfoundland & Labrador Medical Association - Northwest Territories Medical Association - Yukon Medical Association - Society for Rural Physicians of Canada	- Public Health Agency of Canada - Status of Women Canada (renamed Women & Gender Equality Canada in December 2018) - Department of Justice Canada - Health Canada - Mental Health Commission of Canada - Crown-Indigenous Relations & Northern Affairs - Formerly: Ministry of Indigenous Services - Health Canada/ Indigenous Health - Truth & Reconciliation Commission of Canada - National Inquiry into Missing & Murdered Indigenous Women & Girls - Canadian Human Rights Commission - Department of Justice Canada - Victims Services (within Justice Canada)	*Alberta* - Health Services - Ministry of Culture, Multiculturalism & Status of Women - Myhealth.alberta.ca - Ministry of Children’s Services - Ministry of Community & Social Services - Ministry of Justice & Solicitor General *British Columbia* - Ministry of Health Services - Ministry of Children & Family Development - Minister’s Advisory Council on Indigenous Women - Ministry of Public Safety & Solicitor General - First Nations Health Authority - Ministry of Mental Health & Addiction *Manitoba* - Health & Seniors Care - Status of Women Secretariat - First Nations Health & Social Secretariat - Department of Families - Department of Justice, - Department of Indigenous Reconciliation & Northern Relations *New Brunswick* - Department of Health - Department of Social Development - Department of Justice & Office of the Attorney General - Department of Aboriginal Affairs - Women’s Equality Branch - Ministry of Public Safety *Newfoundland & Labrador:* - Department of Health & Community Services - Department of Justice & Public Safety - Ministry of Children, Seniors, & Social Development - Office for the Status of Women *Northwest Territories* - Health & Social Services, - Department of Justice *Nova Scotia* - Department of Health & Wellness - Department of Justice - Department of Community Services - Advisory Council on the Status of Women *Nunavut* - Department of Health - Department of Family Services - Department of Justice - Status of Women Council *Ontario* - Ministry of Health & Long-Term Care - Ministry of Children, Community & Social Services - Ministry of Women’s Issues - Indigenous Affairs - Ministry of the Solicitor General *Prince Edward Island* - Department of Health & Wellness - Department of Family & Human Services - Department of Justice & Public Security - Advisory Council on the Status of Women *Québec* - Ministère de la Santé et des Services sociaux - Ministry of Families, Seniors & the Status of Women - Ministry of Justice - First Nations of Quebec & Labrador Health & Social Services Commission *Saskatchewan* - Health Authority - Ministry of Social Services - Ministry of Justice *Yukon* - Department of Health & Social Services - Department of Community Services - Women’s Directorate - Department of Justice	- Canadian Academy of Child & Adolescent Psychiatry - Canadian Academy of Geriatric Psychiatry - Canadian Academy of Psychiatry & the Law - Canadian Association of Emergency Physicians - Canadian Paediatric Society - Canadian Psychiatric Association	- Center for Research & Education on Violence against Women & Children - FREDA Centre for Research on Violence Against Women & Children - RESOLVE network - Muriel McQueen Fergusson Centre for Family Violence Research - Le centre de recherche interdisciplinaire sur la violence familiale et la violence faite aux femmes (CRI-VIFF) - Centre for the Study of Social & Legal Responses to Violence	- Women’s Shelters Canada - Canadian Shelter Transformation Network - Ontario network of sexual assault/ domestic violence treatment centres - BC Society pf Transition Houses - Ontario Federation of Indigenous Friendship Centres - Ending Violence Association of Canada - Women’s College Hospital Violence & Health Research Program - Alberta Public Health Association - Public Health Association of British Columbia - Manitoba Public Health Association - Public Health Association of New Brunswick & Prince Edward Island - Newfoundland & Labrador Public Health Association - Northwest Territories & Nunavut Public Health Association - Public Health Association of Nova Scotia - Ontario Public Health Association - Saskatchewan Public Health Association - Centre for Addictions & Mental Health	- Canadian Women’s Foundation - Native Women’s Association of Canada - Saskatchewan Towards Offering Partnership Solutions (STOPS) to Violence - Canadian Centre for Child Protection - YWCA Canada - Child Welfare League of Canada - Association of Alberta Sexual Assault Services - Inuit Tapiriit Kanatami - Assembly of First Nations - Métis Nation of Canada - White Ribbon - Immigrant & Refugee Communities Neighbours, Friends & Families - Coalition of Provincial & Territorial Advisory Councils on the Status of Women

**Table 2.** Search Terms Used for Environmental Scan.

IPV search terms		Child maltreatment search terms		Resource search terms
(“intimate partner violence” OR “intimate partner abuse” OR “domestic violence” OR “domestic abuse” OR “battering” OR “dating violence” OR “dating abuse” OR “violence against women” OR “gender-based violence”)	OR	(“child abuse” OR “child neglect” OR “child mistreatment” OR “child endangerment” OR “child * abuse” OR “child exposure to IPV” OR “child exposure to domestic violence” OR “family violence”)	AND	(“curriculum” OR “webinar” OR “resource” OR “training” OR “education” OR “workshop” OR “manual” OR “guide” OR “handbook” OR “tool”)

*Note*. IPV = intimate partner violence.

**Table 3.** Resources Included in Study Analysis.

Resource	Producer	Resource type	Resource format:	Release year	Audience
Intimate Partner Violence	Canadian Psychiatric Association	Education material	Practice guidelines	2012	Practitioners
Domestic Violence Prevention & Reduction In British Columbia (2000–2010)	The FREDA Centre for Research on Violence Against Women & Children	Policy document	Report	2011	Policy makers
Intimate Partner Violence Position Statement & Best Practice Recommendations	Canadian Orthopaedic Association	Education material	Practice guidelines	2019	Practitioners
IPV Consensus Statement	Society of Obstetricians & Gynaecologists of Canada	Education material	Practice guidelines	2005	Practitioners
Woman Victims of Abuse Protocols	Women's Equality Branch, Government of New Brunswick	Policy document	Report	2014	Policy makers
Standards Of Care: Ontario Network Of Sexual Assault & Domestic Violence Treatment Centres	Ontario Network of Sexual Assault/Domestic Violence Treatment Centres	Education material	Practice guidelines	2014	Practitioners, administrators
What The Health Care Community Can Do About Family Violence: Booklet	Government of Alberta	Education material	Summary pamphlet	2008	Practitioners
Intimate Partner Violence: Broaching a sensitive topic with patients	College of Physicians & Surgeons of Ontario	Education material	Newsletter article	2019	Practitioners
Health Effects Of Family Violence	National Clearinghouse on Family Violence	Education material	Summary pamphlet	2003	Practitioners
Emergency Medicine: Key Features of the Priority Topics for the Assessment of Competence In Family Medicine at the Enhanced Skills Level	College of Family Physicians of Canada	Education material	Curriculum document	2017	Practitioners
IPV Systematic Review Summary	VEGA Project	Education material	Practice guidelines	2019	Practitioners, administrators
Healthy Babies, Healthy Families: Postpartum & Postnatal Guidelines	Nova Scotia Department of Health	Education material	Practice guidelines	2003	Practitioners, administrators
Recommendations From The Domestic Violence Death Review Committee	The Domestic Violence Death Review Committee, Department of Public Safety, Chief Coroner’s Office, Government of New Brunswick	Policy document	Report	2014	Policy makers
When She Tells You About The Violence: Tips For General Practitioners	Battered Women's Support Services	Education material	Summary pamphlet	2018	Practitioners
What The Health Care Community Can Do About Family Violence: Information Sheet	Government of Alberta	Education material	Fact sheet	2008	Practitioners
Trauma & Violence-Informed Approaches To Policy Practice	Public Health Agency of Canada	Education material	Guidebook	2018	Practitioners, policymakers
Overcoming Barriers & Enhancing Supportive Responses: The Research On Sexual Violence Against Women A Resource Document	Centre for Research & Education on Violence against Women & Children	Education material	Literature review	2012	Practitioners
Document, Monitor, Collaborate: A Primer on Domestic Violence Risk Assessment & Management	Center for Research & Education on Violence against Women & Children	Education material	Video module	2016	Practitioners
Suffering In Silence: An Assessment Of The Need For A Comprehensive Response To Sexual Assault In Nova Scotia	Nova Scotia Sexual Assault Services Planning Group; endorsed by Department of Community Services, Nova Scotia	Policy document	Report	2008	Policy makers
Trauma-Informed Practice In Different Settings & With Various Populations – A Discussion Guide For Health & Social Service Providers	Nova Scotia Health Authority, IWK Centre	Education material	Training guide	2015	Practitioners
Providing Trauma-Informed Care to 2SLGBTQ + Patients	Native Women's Association of Canada	Education material	Fact sheet	2019	Practitioners
Transforming Our Response To Sexual & Reproductive Health	Native Women's Association of Canada	Education material	Fact sheet	2018	Practitioners, administrators
Hospital Guidelines For The Treatment Of Persons Who Have Been Sexually Assaulted (3rd Edition)	Ontario Hospital Association	Education material	Practice guidelines	2018	Practitioners, administrators
3 Considerations For Supporting Women Experiencing Intimate Partner Violence During The Covid-19 Pandemic	Centre for Research & Education on Violence against Women & Children	Education material	Fact sheet	2020	Practitioners
A Strategic Framework To In New Brunswick End Violence Against Wabanaki Women	New Brunswick Advisory Committee on Violence against Aboriginal Women	Policy document	Report	2008	Policy makers
Dealing with Distress from Patients’ Trauma	Alberta Medical Association	Education material	Newsletter article	2010	Practitioners
Why VEGA? Video	VEGA Project	Education material	Video	2019	Practitioners
Intimate Partner Violence in a Pandemic	Centre for Research & Education on Violence against Women & Children	Education material	Fact sheet	2020	Practitioners

### Eligibility

We defined an “IPV resource” as a policy document or training material focused on describing or developing knowledge, attitudes, skills, or behaviors that a physician should possess related to IPV. For inclusion in this analysis, IPV resources had to: (a) have been released or revised since 2000 and be available in English; (b) be accessible for free online, without requiring registration; (c) be for a Canadian audience, reflected by having been produced or explicitly endorsed by any of the Canadian organizations included on our initial search list; and (d) be complete enough to facilitate the present discourse analysis (e.g., we excluded slide decks from presentations where the presenter or the intended audience were unclear, or where audio recordings of the presentation accompanying the slides were not available).

### Data Extraction

As resources were provisionally identified, we used a data extraction form (Appendix 2) to record key details about each resource. We collected the name of each resource, the URL at which it was collected, the organizations involved in producing and disseminating it, the region on which it focused (if any), the date it was released and last updated on, the modes of content delivery it employed, any specific target audiences it identified, as well as specific patient populations centered within the resource. This data served two purposes: to help determine whether a given resource met the criteria for inclusion in the present study and to generate a “face sheet” for each resource that provided context during analysis. All resources that were identified that met the inclusion criteria were imported into N-Vivo: text-based resources were included as PDFs, while video and multimedia resources were transcribed, and annotated with descriptions and screenshots of on-screen imagery and figures.

### Data Analysis

We used CDA to analyze our data. CDA considers how “hegemonic power relations are discursively produced, sustained, negotiated, and challenged in different contexts and communities” (Lazar, 2007). Close critical analysis of texts, images, and other discursive formations afford insight into the ways that communications are constitutive of social power dynamics (Kuper et al., 2013; Rogers et al., 2005). In all discourse, but particularly in the context of resources intended to shape medical practice, claims to truth and legitimacy have material effects in the world. For example, in the context of this work, claims and directives about how physicians *should* address IPV in their medical practice works to structure what forms of care and aid are accessible to those experiencing IPV.

In keeping with principles of CDA, our coding framework evolved iteratively through group discussion: an initial reading of the resources, informed by a review of relevant literature, was used by AC to develop a formative codebook covering a broad range of areas of potential interest. Three coders (AC and two research assistants) subsequently coded and re-coded the resources to refine these codes before AC revised the codebook to its final form focusing on the question “how are physicians’ roles represented in training materials about IPV for a medical audience?” In this final stage of analysis, we identified excerpts from resources that implicitly or explicitly offered normative guidance related to IPV for physicians. AC then inductively grouped and regrouped these codes until a final model encompassing all of the roles physicians play in addressing IPV was realized.

## Results

We reviewed more than 22,000 search results and identified 28 resources that met inclusion criteria for the present analysis. These resources were produced by 22 organizations between 2003 and 2020 (see Table 4 for a complete list of included resources); 23 resources were education materials with a primary audience of practitioners and/or health care administrators and five were reports with a primary audience of health policymakers. Of the resources for practitioners, most were targeted for a general audience of physicians and other health care providers; four targeted physicians from specific specialties—namely, family medicine, emergency medicine, orthopaedic surgery, and psychiatry. The majority of resources we identified (26) were text based, including practice guidelines (7), reports (5), fact sheets (5), pamphlets (3), newsletter articles (2), and other miscellaneous training resources (4); one multimedia module and one video were also identified.

**Table 4.** Summary of Physician Roles in Addressing Intimate Partner Violence (IPV).

Role	Description	Examples
A. Learning	Physicians are expected to engage in ongoing, self-directed *learning* about IPV in preparation for performing the *identifying* and *responding* roles.	- “Health care providers are encouraged and supported to engage in ongoing professional activities including educational opportunities, case consultations and peer review sessions to maintain skill and competency levels.” (Ontario Network of Sexual Assault/Domestic Violence Treatment Centres, 2014) - “These strategies include education and training for health care professionals to help them recognize the signs of violence and abuse and to respond sensitively, respecting the diverse needs of victims from vulnerable population groups.” (Rossiter, 2011) - “Learn how to spot the signs of family violence in all its forms, such as physical abuse, psychological abuse, sexual abuse and neglect. Make family violence education part of your ongoing professional development. If you lack experience in this area, seek opportunities to gain experience.” (Alberta Children’s Services, 2008a)
B. Identifying	Physicians are expected to *identify* patients affected by IPV by perceiving signs that may be suggestive of abuse, and by asking patients directly if they are experiencing IPV.	- “The role of emergency room staff includes detection of women who are victims of intimate partner violence” (Woman Victims of Abuse Protocols, 201*4*) - “It is especially important for mental health clinicians to be alert to the signs and symptoms of IPV exposure, and to practice case finding for IPV in the assessment of patients who present with psychological signs or symptoms (such as depression, anxiety disorders, including PTSD, chronic pain, eating disorders, sleep disorders, psychosomatic disorders, self harm, substance abuse, some personality disorders, and nonaffective psychosis) or physical signs or symptoms (see above), which are known to be associated with IPV exposure.” (Stewart et al., 2013)
C. Responding	Physicians are expected to *respond* to patients who disclose IPV or who they suspect are experiencing IPV, by providing emotional support, medical treatment, and referrals to community resources, by assessing patient safety, by documenting in the patient’s medical record, and by reporting concerns related to child maltreatment if they arise.	- “If abuse is confirmed, your immediate goals are to ensure the person is safe and to provide support. (Tell them, ‘It is not your fault. You deserve to be safe. Help is available.’)” (Alberta Children’s Services, 2008b) - “Essential elements of health sector response include documentation, risk assessment, addressing the safety of children present in the home, facilitation of a safety plan, and effective referral and follow-up.” (Cherniak et al., 2005) - “When abuse is suspected, or confirmed, ensure that management includes the patient’s informed consent and agreement to the plan, reports to authorities as appropriate, and a disposition that ensures the safety of the patient and other vulnerable parties (e.g., children, elders).” (College of Family Physicians of Canada, 2017)

**Figure 1.**
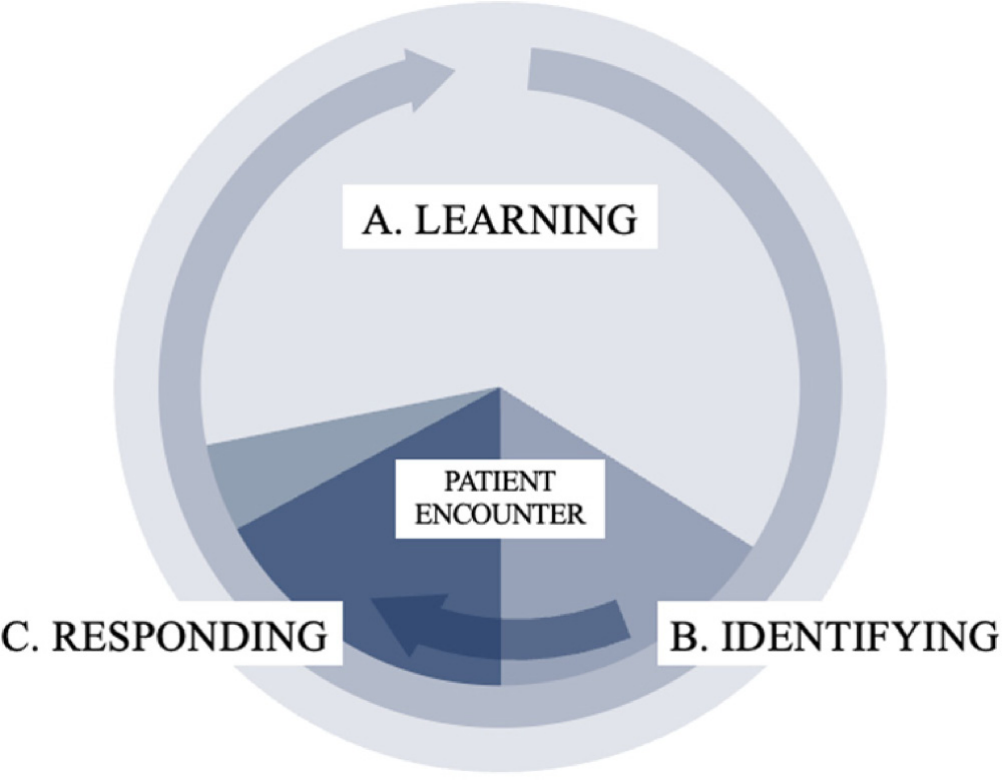
Overview of physician roles in addressing intimate partner violence (IPV).

In analyzing these materials, we identified three connected roles that physicians are expected to perform related to IPV: *learning* about IPV, *identifying* patients experiencing IPV, and *responding* to patients’ disclosures of IPV. The cyclical relationship between these roles is shown in Figure 1. The *learning* role includes both formal education and training experiences and self-directed learning in independent practice. Physicians are expected to learn continually so that they are primed to identify and respond to patients in their care who are experiencing IPV. The *identifying* role, by contrast, is performed in the context of encounters with patients and/or their family members as physicians learn or suspect their patients are affected by IPV. Once patients are identified, the *responding* role is enacted during patient encounters, as physicians provide support and referrals, and continues afterward, as physicians document their interactions with patients in their medical records and discharge their legally mandated reporting obligations if they have concerns related to child maltreatment.

### Learning

Learning about IPV, also formulated passively as “knowing” about IPV, is represented as a core, ongoing expectation for all physicians in these resources. Physicians are expected to learn about IPV before, during, and after they participate in the care of patients they know or whom they suspect to be affected by violence. Starting in their undergraduate medical education, (Rossiter, 2011; Stewart et al., 2013), being knowledgeable about the nature, prevalence, and sequelae of IPV is constructed as necessary for “good medical practice” (Canadian Orthopaedic Association, 2019). As training progresses into residency, expectations around learning evolve, encompassing familiarity with community resources and information related to patient care that is contextually relevant to the medical discipline in which they practice (Cherniak et al., 2005; Stewart et al., 2013). Physicians are expected to identify deficits in their knowledge related to IPV and “pursue professional development opportunities to gain necessary skills” in a self-directed manner (*Woman Victims of Abuse Protocols, 2014*). Attending training courses for continuing professional development, seeking out case consultations, and participating in peer reviews with colleagues are all described as potential opportunities to learn (Ontario Network of Sexual Assault/Domestic Violence Treatment Centres, 2014).

Almost all of the resources considered in this analysis portray the central motivations for physicians to learn about IPV as increasing their capacity to recognize signs and symptoms of abuse, and their preparation to solicit and respond to disclosures of IPV. Honing these active skills is described as an intervention that may “save a life” (Alberta Children’s Services, 2008a) and as a “lifeline” (“Intimate Partner Violence: Broaching a sensitive topic with patients,” 2019) to patients experiencing abuse. By contrast, contextual or theoretical knowledge about IPV is positioned as less central to medical practice: although several of the resources describe “risks” or “risk factors” that increase the likelihood of experiencing IPV (Doherty, 2003; Woman Victims of Abuse Protocols, 2014), these risk factors are presented with limited discussion of the mechanisms and contextual factors that explain how these risks are produced.

### Identifying

While learning about IPV is represented as an ongoing preparatory physician role that occurs outside the immediacy of patient care, identifying and responding to IPV are constructed as action-oriented roles that are largely operationalized during encounters with an individual patient. Perceiving and asking about IPV are presented as central, related components of the identifying role, encompassing recognizing patient “presentations that may be suggestive of undeclared abuse” (College of Family Physicians of Canada, 2017) and directly asking patients whether they are experiencing IPV. These facets of the role are coupled in most of the more recent resources (VEGA Project. 2016), reflecting current guidance *against* universal screening for IPV; earlier resources are more equivocal about the use of screening (Domestic Violence Death Review Committee, 2015; Healthy Babies, Healthy Families: Postpartum and Postnatal Guidelines, 2003).

“Perceiving” is variously described in these resources, in some instances, passively, as being “sensitive” (Cherniak et al., 2005) or “aware” (Doherty, 2003) of signs and symptoms of abuse, and in others, actively, as “detecting” (Alberta Children’s Services, 2008a; Battered Women’s Support Services, 2019; Woman Victims of Abuse Protocols, 2014) patients experiencing violence. Resources note that physical injuries may be indicative of violence, but that patients may try to hide or offer alternative explanations for how these injuries arose (Alberta Children’s Services, 2008b; Cherniak et al., 2005; VEGA Project. 2016). Beyond physical injuries, the signs and symptoms of IPV that these resources discuss are often vague. Anger, sadness, anxiety, fear, and fatigue are all described as “emotional signs” of IPV (Alberta Children’s Services, 2008b). A wide array of chronic physical health concerns (Baker et al., 2012; Public Health Agency of Canada, 2018), as well as drug and alcohol abuse (Centre for Research and Education on Violence against Women and Children, 2021), are also noted as indicators. Although perceiving abuse is largely portrayed as an attentiveness to presenting signs or symptoms, and observations of the patient, the perceiving role also includes observation of interactions with others accompanying the patient, including a partner and children (Rubin, 2008). Certain care-seeking behaviors—delaying care, or missing appointments—are also noted as possible indicators of abuse (Alberta Children’s Services, 2008b; “Intimate Partner Violence: Broaching a sensitive topic with patients,” 2019).

Reflecting the nature of IPV as a gendered form of violence, the signs and symptoms of IPV described in these resources are often framed, either explicitly or implicitly, in relation to gender. For instance, a provincial protocol for “Women Victims of Abuse” includes parallel lists of signs that a man is being abusive and that a woman is experiencing abuse that feature many of the same entries on both lists (Woman Victims of Abuse Protocols, 2014). Gender, in this instance, is produced as the key explanatory variable that instructs physicians in interpreting their patient’s behavior as indicative of “being abusive” versus “experiencing abuse.” The physician’s role focusses on identifying victims of violence (usually female); notably, any role physicians might play in identifying people who are violent to their partners, or in supporting men who are victims of IPV regardless of the gender of their partner, pass largely undescribed. Gender nonconforming people are centered in only one resource with a specific focus on queer and trans communities (Native Women's Association of Canada, 2019).

Asking about IPV either based on presenting signs, symptoms, and potential risk indicators (sometimes referred to as case-finding) or using universal screening, is described in nearly all of the resources we examined. Approaches to asking about IPV are carefully delineated in both affective terms—physicians should ask “without pressure” (Trauma-informed practice in different settings and with various populations: A Discussion Guide for Health and Social Service Providers, 2015), and “non-judgementally” (College of Family Physicians of Canada, 2017; Native Women's Association of Canada, 2019)—and in procedural terms—ask “routinely” (Woman Victims of Abuse Protocols, 2014), or with the use of a validated screening tool (Canadian Orthopaedic Association, 2019; Domestic Violence Death Review Committee, 2015). Several resources identify pregnant patients as particularly vulnerable to IPV, necessitating increased attentiveness to these concerns on the part of physicians (Cherniak et al., 2005; Ontario Network of Sexual Assault/Domestic Violence Treatment Centres, 2014; Canadian Orthopaedic Association, 2019). Examples of specific language and phrasing to use while asking about violence abound (Battered Women’s Support Services, 2019; Native Women's Association of Canada, 2018, 2019), and several resources also include follow-up replies that help direct the conversation toward initiating the *responding* role. Spontaneous disclosure of IPV by a patient is rarely described in these resources (Cherniak et al., 2005). Contextual factors—including trust in the patient‒provider relationship, and privacy in the clinical environment—are described as necessary for facilitating disclosure, either unprompted or in response to a provider’s questioning (Canadian Orthopaedic Association, 2019; Cherniak et al., 2005; Stewart et al., 2013).

In these resources, the act of asking about IPV is represented as serving a dual purpose: it creates opportunities for patients to disclose experiences of IPV, while also making clear to patients that IPV falls within the clinician’s scope of practice. This discursive function of asking about IPV is recognized explicitly in these resources: the Canadian Orthopaedic Association states explicitly that part of the value of asking patients about IPV is “convey[ing] that health care professionals view IPV as an important health issue and that they are open to discussing it and providing assistance” (Canadian Orthopaedic Association, 2019). Asking about violence, in the clinical context, figures as a form of action unto itself, albeit one that necessarily demands further response.

### Responding

Following immediately after a patient is identified, or identifies, as experiencing violence, “responding” is constructed as the subsequent role physicians play in addressing IPV. “Responding” encompasses several interconnected actions, such as providing support, assessing and making plans to address immediate safety concerns, assessing patients’ mental and physical health and social needs, and either providing treatment to patients directly or making referrals to other clinicians and to community resources. Part of the physician’s role also includes actions related to legal responsibilities, most commonly described as documenting information in patients’ medical records and making mandated reports to child protection agencies or to police where indicated.

Resources reviewed in this analysis situate physicians’ immediate response to disclosures of IPV as critically important to establishing “an effective therapeutic relationship” (College of Family Physicians of Canada, 2017). In their response, physicians are instructed to be “non-judgemental” (Canadian Orthopaedic Association, 2019) and “validating” (Public Health Agency of Canada, 2018; Woman Victims of Abuse Protocols, 2014), reinforcing that whatever violence a patient may experience is not their fault; clinicians are also enjoined to offer their support “unconditionally” (Ontario Hospital Association, 2018; Ontario Network of Sexual Assault/Domestic Violence Treatment Centres, 2014) and to center “the patient’s individual concerns and decisions” (Stewart et al., 2013). In cases where abuse is suspected, but patients deny violence is an issue, physicians are instructed to respect patients’ decisions not to disclose it. Indeed, showing respect for patients’ autonomy is represented as centrally important to providing a “safe” (Baker et al., 2012) response to a disclosure of IPV across the resources reviewed for this analysis: physicians are instructed to clearly delineate the limits of doctor-patient confidentiality, and to obtain informed consent from patients before making referrals except where mandated by law (Baker et al., 2012; Trauma-informed practice in different settings and with various populations: A Discussion Guide for Health and Social Service Providers, 2015). Physicians are encouraged to problematize and define the interpersonal violence in their patients’ lives both passively, in the form of posters and reading material in their offices, and actively, in the form of “offer[ing] information” (Doherty, 2003) and “emphasizing the unacceptability of violence” (Battered Women’s Support Services, 2019). Similarly, in the resources reviewed in this analysis, graphic representations of the process of responding to IPV suggest unidirectional pathways between disclosures of violence, safety planning, and referrals to community resources, offering little guidance to physicians in caring for patients who are more ambivalent about, or who outright reject, their attempts to offer intervention (Alberta Children’s Services, 2008a; Battered Women’s Support Services, 2019).

After providing immediate emotional support to patients, physicians are instructed to assess the patient’s mental or physical health concerns and offer either treatment for those concerns or referrals as appropriate given their scope of medical practice. Referring patients to “community resources” figures prominently in this facet of the responding role (Cherniak et al., 2005; Healthy Babies, Healthy Families: Postpartum and Postnatal Guidelines 2003; Woman Victims of Abuse Protocols, 2014), but seldom are the nature of these resources or the extent of assistance they can provide described. Conducting or facilitating a safety assessment to appraise any imminent risk to a patient is also featured as a core component of responding to disclosures of violence, with resources providing widely varying degrees of detail (3 Considerations for Supporting Women Experiencing Intimate Partner Violence During the COVID-19 Pandemic 2020; Alberta Children’s Services, 2008a). Making plans to follow up with the patient, and clearly charting the encounter and results of the safety assessment, are the final features of this role described in these resources. Documenting is framed in terms of producing evidence that “could be of benefit to the patient sometime in the future” (Canadian Orthopaedic Association, 2019), and physicians are encouraged to describe any violence mentioned by a patient with specificity and objectivity, avoiding any personal editorializing (Alberta Children’s Services, 2008a; Cherniak et al., 2005). Scheduling follow-up with a patient is portrayed as the natural conclusion to the responding role, proffering an opportunity to appraise whether IPV has escalated, and to see whether patients have followed through on accessing supports or any referrals.

## Discussion

Our analysis found that resources for a medical audience consistently constructed three interconnected roles for physicians in addressing IPV. On an ongoing basis, physicians are expected to *learn* about IPV, in order to *identify* patients they provide care for who are affected by IPV, and then to *respond* to these patients in a wide array of ways.

The construction of these aspects of the physician role, as reflected in the resources reviewed for this paper, bear the hallmarks of a process of medicalization. Medicalization describes a process through which social and political facets of “everyday life” are redefined in relation to illness and health; through this process, broad swaths of human experience come to be understood as objects of medical expertise, and are thereby made subject to medical intervention (Illich, 1975). In the context of IPV, medicalization has been critiqued for concealing the varied structural oppressions implicated in producing trends related to IPV victimization and perpetration observed at a population level (Nandakumar, 2018; Sweet, 2015; Wilkerson, 2018). Centering the public health impacts of violence has strategic political value in shaping resource allocation, but biomedical discourses that present socially mediated identity categories as “risk factors” for violence naturalize power inequities, and render resources and support less accessible to communities for whom health care spaces are hostile or unsafe (Sweet, 2015).

Physicians play an important role in medicalization, helping, not only, to address or “solve” their patients’ medical problems, but also to frame how these problems are conceived of by patients themselves. Schön describes this “problem-setting” as a function of professionalization in many settings, that enables professionals to “impose … a coherence which allows [them] to say what is wrong and in what direction the situation needs to be changed” (Schön, 1983, p. 40). This process of “naming” and “framing” problems is foundationally discursive and is shaped, inevitably, by physicians’ own subject positions, values, ideologies, and privileges (Ritz, 2015). In clinical settings, physicians typically control the discourse and are able to assert their preferred framing of an issue save in circumstances where patients are either very persistent or persuasive (Kinsella, 2006). Through this process of medicalization, physicians are empowered to impose an understanding of IPV that is “matched to their professional knowledge and know-how” on their patients, channeling their response accordingly (Schön, 1987, p. 36).

Evidence reflecting an ongoing process of medicalization of IPV can be seen in the following features of these resources: physician roles in addressing IPV are constructed as active and interventionist; IPV is problematized as a health issue that can be effectively addressed in a medical setting; physicians are positioned as having the professional authority and medical knowledge to “educate” patients about IPV (Alberta Children’s Services, 2008a; Baker et al., 2012). At times, the impetus toward physician intervention that animates these resources operates even in contravention of best medical evidence. For example, although research examining the impacts of routine IPV screening suggested limited or no benefits to patients as early as 2001 (O’Doherty et al., 2014), instructions outlining an approach to “family violence screening in emergency departments” in the absence of any concerning signs or symptoms is featured prominently in two resources published in 2008 (Alberta Children’s Services, 2008a). Even later resources—including, more concerningly, best practice recommendations published in 2019—also advocated physicians take a proactive approach to identifying patients experiencing violence: orthopedic surgeons are encouraged to “routinely ask all female patients about IPV” (Canadian Orthopaedic Association, 2019), despite strong evidence underscoring the lack of benefit from universal screening (Feltner et al., 2018).

These examples, where a medicalized push toward intervention seems even to overstep medicine’s own epistemological claims to authority are striking. Other examples are more insidious. For example, although the responding role is constructed as encompassing both passive supportive elements and action-oriented referrals, the active facets of this physician role are centered across many of these resources. Physicians’ medico-legal responsibilities related to reporting suspected child maltreatment feature prominently in many resources, as do inducements to physicians, to “reframe” violence for their patients as unacceptable or untenable, producing their epistemic authority over their patients’ own experiences. In one resource, of fourteen bullet points describing “issues to keep in mind when addressing IPV” eleven describe specific actions a physician should take (IE: risk assessment, safety planning, documenting, arranging for follow-up, providing referrals) (“Intimate Partner Violence: Broaching a sensitive topic with patients,” 2019); in another, after a patient discloses IPV, a suggested expression of support is “there are things we can discuss that can help” (Stewart et al., 2013). Although affective guidance—related to the tone a physician should strike, or the type of environment they should foster for patients—is abundant, these instructions, which address what many physicians report finding most difficult about responding to IPV, are often vague one-liners. None of the resources reviewed in this analysis offer specific examples of what a physician might say if a patient denies suspected abuse or how to respond when a patient chooses not to pursue a proffered referral; likewise, although affirming the agency of patients who decide to stay in violent relationships is constructed as centrally important to empowering patients, what support looks like in this context is left to physicians to construe on their own.

The process of responding by “referring” is left similarly open-ended. It is suggested that physicians “have a list of local resources and support information on hand” but the nature or constraints of the supports implicitly presumed available are rarely described (“Intimate Partner Violence: Broaching a sensitive topic with patients,” 2019). In part, this reflects a practical limitation: available referrals of the manner of those mentioned in these resources are contingent on location, and other factors, and policies impacting the types of services available can vary widely. This vagueness also serves to obscure political realities—linked to both the medicalization and criminalization of IPV by way of policy choices governing the allocation of limited public funding—that circumscribe what community resources do exist to address the needs of people experiencing violence. Presupposing the availability of broadly termed “community resources” belies waitlists for publicly funded counselling, and limitations on how people and families experiencing violence can access emergency shelters; it also obscures larger holes in the social safety net related to access to legal counsel and to financial support, and governed by immigration status, that may discourage people from leaving violent relationships.

While this analysis has primarily focused on parsing physician roles in addressing IPV, other subject positions are also, necessarily, produced in these resources as well. People experiencing violence are represented, primarily, in their role as patients or as parents, omitting other roles they may also occupy in their families and in their communities. The assemblage of “risk factors” and health outcomes associated with the “condition” of experiencing violence are made central to patients’ identities, while communities are mentioned as requiring special “cultural considerations” for assessment or treatment or most at risk of being affected by violence are implicitly portrayed as being especially violent. Given the role physicians play as mandated reporters of suspected child maltreatment—increasingly defined to include children’s exposure to IPV—the implications of increased suspicion toward communities that are singled out via “culture” can have serious consequences for families.

This is not to say that physicians should not learn about how “culture” is implicated in shaping patterns of violence and abuse, but rather that careful attention must be paid to how culture and context are framed in these resources. Attending to structural forces that give rise to marginalization and disparities in exposure to violence, and in health and wellbeing more broadly, enables physicians to better appreciate proximal opportunities to address patients’ immediate safety and health concerns, as well as distal opportunities for political advocacy and solidarity with communities’ organizing efforts (Metzl & Hansen, 2014). It also opens up space for physicians to recognize their own biases, and to more empathetically engage with patients affected by violence (Mackenzie et al., 2019).

In addition and in contrast with the “abused patient’s” hyper-visibility, the role of people who commit IPV is largely peripheral to the medical encounters imagined in these resources. Defined by their male gender, their overbearing presence, and their risk of committing future violence, people who are violent in relationships are not portrayed in these resources as patients whom physicians might encounter on their own. Finally, patients who have experienced IPV in the past are also largely absent from these resources. Although evidence is included in virtually all these resources that describes the enduring health effects of IPV even after patients have left violent relationships, the principles for caring for people with historical trauma related to violence is not a primary focus in these materials.

### Strengths and Limitations

This study’s main strengths lie in its novel application of environmental scanning and CDA methodologies, and is the first study we are aware of to use a systematic approach to assemble and analyze training resources that address IPV for a medical audience; similarly, it is the first study we could find that used CDA to parse how IPV is represented in the medical education context. Limitations of our study relate to challenges in assembling a truly comprehensive picture of the Canadian IPV resource landscape: our methodology did not seek to determine which of the resources we reviewed were most influential in the medical milieu, or which are less commonly made use of by practitioners or policy makers to shape medical practice; it also did not seek to evaluate resources’ use of evidence-based pedagogical approaches or their impacts on physician knowledge, attitudes, skills, and behaviors. Additionally, our dataset did not include resources that required registration. Although this was by design—we wanted to see what was most readily available to practitioners seeking to bolster their skills and knowledge related to IPV—other forums for training related to IPV that shape the practice of Canadian physicians include in-person training and online training that requires registration or specialized access.

## Conclusion

This study has considered how the roles that physicians play in addressing IPV are constructed in resources for Canadian physicians. Combining environmental scanning methodology with CDA, we identified three connected physician roles—learning about IPV, identifying patients experiencing IPV, and responding to patients’ disclosures of IPV—that were produced in resources with a medical audience. Our conjecture is that these formulations of the physician role, and of IPV itself, reflect a process of medicalization; physicians are instructed to adopt an interventionist stance in addressing IPV, and encouraged to frame IPV in their own and in their patients’ understandings, as a health issue that can be effectively addressed in a medical setting. This process of medicalization has material implications not only for providers, but most significantly for patients structuring what resources and support are accessible to those experiencing IPV.

## Appendix

### Appendix 1: Hand Search Tracking Sheet

**Table:**

Searcher Initials	Search Date	Organization	Resource URL	Path Resource Identified	Resource Inclusion Criteria	Include Resource?	Additional Notes
	DD/MM/YY	*Name of Organization from Master List*	*Permalink to Resource*	*Check Applicable*	*Check Off all Criteria that Apply*	*Y/N/Borderline*	*Include any further comments*
				□ Directly through Google Search	□ Addresses Family Violence (defined as IPV, CM, or CEIPV)		
				□ Through a Webpage (w/ Linked Resources)	□ Addresses audience that includes MDs		
				□ Resource Requiring Registration	□ Developed/adapted for Canadian audience since 2000		
					□ Produced/explicitly endorsed by organization		

### Appendix 2: Data Extraction Form

Resource Name:

Resource URL:

Organization(s):

(Note the name or names of the organization or organizations involved in producing the resource)

Region:

IE: National, Provincial—Ontario, Regional—Toronto

Release Date:

(Include Year and Month if Available)

Last Updated:

(Include Year and Month if Available)

Mode(s) of Content Delivery:

(Check all that apply)

◻ Video Vignettes
◻ Training Manual
◻ Infographic
◻ Report
◻ Webinar
◻ PowerPoint Presentation

Other:

Target Audience(s), if stated:

Communit(ies) of Focus, if any:

Date Accessed:
